# pH Manipulation as a Novel Strategy for Treating Mucormycosis

**DOI:** 10.1128/AAC.01366-15

**Published:** 2015-10-13

**Authors:** Wioleta J. Trzaska, Joao N. Correia, Maria T. Villegas, Robin C. May, Kerstin Voelz

**Affiliations:** a Institute of Microbiology and Infection and School of Biosciences, University of Birmingham, Birmingham, United Kingdom; b NIHR Surgical Reconstruction and Microbiology Research Centre, University Hospitals of Birmingham NHS Foundation Trust, Queen Elizabeth Hospital Birmingham, Birmingham, United Kingdom

## Abstract

Mucormycosis is a fatal fungal disease caused by several organisms within the order Mucorales. In recent years, traumatic injury has emerged as a novel risk factor for mucormycosis. Current antifungal therapy is ineffective, expensive, and typically requires extensive surgical debridement. There is thus a pressing need for safe prophylactic treatment that can be rapidly and easily applied to high-risk patients, such as those with major trauma injuries. Acetic acid has been used as a topical treatment for burn wounds for centuries and has proven activity against Gram-negative bacteria. Here, we demonstrate that acetic acid is also highly effective against major pathogenic groups of Mucorales, even at very low concentrations (0.3%). This antifungal effect is not seen with other acids, such as hydrochloric and lactic acid, suggesting that acetic acid activity against Mucorales spores is not solely evoked by low environmental pH. In agreement with this, we demonstrate that the antifungal activity of acetic acid arises from a combination of its ability to potently lower intracellular pH and from pH-independent toxicity. Thus, dilute acetic acid may offer a low-cost, safe, prophylactic treatment for patients at risk of invasive mucormycosis following traumatic injury.

## INTRODUCTION

Mucormycosis is an infection caused by fungi that belong to the order Mucorales, pervasive environmental fungi found in soil and decaying wood. Within the class Mucormycetes, the order Mucorales contains the genera Rhizopus, Mucor, Rhizomucor, and Lichtheimia, which cause most cases of human infection (1). Over the past two decades, the frequency of patients with mucormycosis has increased significantly (2). This increase in infections is associated with excessive morbidity and mortality, and it is directly related to an increasing patient population with underlying immunocompromising conditions which put them at risk for the development of serious fungal infections like mucormycosis (3). These include individuals suffering from uncontrolled diabetes mellitus, patients who have received an organ or hematopoietic stem cell transplant, patients with malignancy or neutropenia, and those who receive deferoxamine therapy or intravenous drug users (4, 5). Frequently, however, cases of mucormycosis also occur in immunocompetent patients who have received traumatic injuries in settings such as agricultural accidents, motor vehicle collisions, and blunt crush injures, as well as during natural disasters (6). In addition, several cases have been reported recently in military personnel, secondary to blast injuries caused by improvised explosive devices (7).

Established mucormycosis has an extremely high mortality rate, in excess of 90% (4). Effective treatment of this disease thus depends on an early diagnosis and prompt initiation of therapy. Frontline antifungals used for treatment of mucormycosis include amphotericin B in various liquid formulations and posaconazole (8, 9). Amphotericin B is the more active agent but carries significant risks of organ toxicity. Posaconazole appears to be well tolerated and is recommended to be used for treatment in patients with amphotericin B intolerance or those unresponsive to previous antifungal therapy (10, 11). In high-risk patient groups (such as severely injured military personnel) prophylactic administration of posaconazole prior to diagnosis may be effective, but this is economically unfeasible in most settings. Thus, a cheap, safe, and easy-to-apply prophylactic treatment to reduce the incidence of mucormycosis in open wounds is likely to have significant clinical benefits.

Acetic acid has been used for thousand of years as an antimicrobial agent, for instance, to reduce the spread of plague in medieval times or as a wound dressing in the American Civil War (12). More recently, it has been used to treat burn wound infections, and it has demonstrable activity against several bacterial pathogens (13). To date, however, its activity against fungi in general and mucormycosis in particular has not been tested.

Here, we demonstrate that acetic acid has potent antifungal efficacy at very low concentrations against all Mucorales species tested. This activity is not seen for other acids. Using ratiometric fluorescence reporters, we show that fungal spores treated with acetic acid undergo rapid acidification of their cytoplasm and become arrested without germination.

Thus, we propose that early application of dilute acetic acid may represent an effective and low-cost strategy to minimize mucormycosis in traumatic wounds.

## MATERIALS AND METHODS

### Mucorales strains.

Four different isolates were tested; Lichtheimia corymbifera 9.6002134 and Rhizopus microsporus 12.6652333, which are clinical isolates, and Mucor circinelloides NRRL3631 and Mucor circinelloides CBS277.49 (4). All strains were grown on Sabouraud agar plates for at least 10 days before use.

### Media.

Acetic acid experiments were performed in Sabouraud broth (Fisher Scientific) and complete RPMI medium 1640 (cRPMI), phenol free (Gibco), plus 100 U/ml of penicillin and 100 U/ml of streptomycin (Sigma), 2 mM l-glutamine (Sigma), and 10% fetal bovine serum (FBS) (Sigma) with different concentrations of acetic acid (Tables 1 and 2). For hydrochloric and lactic acid treatments, the pHs in Sabouraud broth and cRPMI were matched with the pHs generated by the different concentrations of acetic acid. Spores were washed with phosphate-buffered saline (PBS), spun down, and resuspended in PBS for cell counting in a hemocytometer. Spore counts were adjusted to a final count of 1 × 10^4^ cells per ml.

**TABLE 1:** pHs for different concentrations of acetic acid in Sabouraud medium

% acetic acid	pH
5	2.52
2.5	3.27
1.25	3.63
0.63	3.92
0.31	4.18
0.16	4.43
0.08	4.68
Sabouraud medium only (control)	5.73

**TABLE 2:** pHs for different concentrations of acetic acid in cRPMI medium

% acetic acid	pH
5	2.32
2.5	3.19
1.25	3.67
0.63	4.03
0.31	4.46
0.16	5.24
0.08	6.32
RPMI medium only (control)	7.45

### Fungal growth inhibition assays.

Five percent acetic acid (Tayside Pharmaceuticals, Dundee, United Kingdom) was serially diluted into Sabouraud medium and cRPMI to produce concentrations of 2.5%, 1.25%, 0.63%, 0.31%, 0.16% and 0.08%. The pH of each of these concentrations of acetic acid was measured, and then pH-matched solutions of hydrochloric acid (Fluka) or lactic acid (Sigma) were produced in Sabouraud and cRPMI media (Tables 1 and 2). For pH-neutralized experiments, media were generated as described above, but then, 1 M NaHCO_3_ was added to return the pH to that of the control. Fungal growth rate was measured by reading the optical density at 600 nm (OD_600_) at time zero and after 24 and 48 h using a microplate reader (BMG Labtech). Plates were incubated at 37°C in between reads, without shaking. To estimate spore killing, samples from cultures with the concentrations of acetic acid in which no growth was observed were plated onto Sabouraud agar plates, and the numbers of CFU were counted 24 h later. Each experiment was performed with at least three experimental replicates and three biological repeats.

### Time-lapse imaging.

Time-lapse imaging was performed on fungal spores either prior to or shortly after germination, using a Nikon Eclipse Ti microscope with a long working distance (LWD) 0.52 20× objective for 18 h. Movies were analyzed and prepared for publication using NIS Elements software.

### Time of action.

To test how rapidly acetic acid affects fungal viability, spores were incubated for 0, 15, 30, 60, 120, 180, 240, and 300 min in 2.5% acetic acid (in cRPMI) and then plated onto Sabouraud plates for subsequent colony counting 24 h later. Scoring for CFU at this early time point requires more careful observation but avoids the problem of extensive filamentation that leads to fused colonies.

### Measurement of pH_i_.

Fungal spores at a concentration of 6 × 10^6^/ml were incubated in RPMI medium at 37°C and 5% CO_2_ for 4 h before imaging. Cells were then incubated with 5 μM BCECF-AM [2′,7′-bis-(2-carboxyethyl)-5-(and-6)-carboxyfluorescein, acetoxymethyl ester; Life Technologies, USA] for 1 h and resuspended in fresh medium to allow full deesterification of the dye. Intracellular pH was measured via single-cell ratiometric imaging at ×60 magnification using an Olympus IX81 (Olympus, United Kingdom) coupled to a monochromator-based illumination system (Cairn Research, United Kingdom) and an Evolve 512 EMCCD (Photometrics, USA) digital camera; image acquisition was controlled using MetaFluor (Molecular devices, USA) acquisition software. Fluorescence emission for excitation wavelengths centered at 490 nm and 436 nm was captured at 530 nm, and ratios were obtained for individual cells after background subtraction. Ratios were converted to intracellular pH values after *in situ* calibration as described by James-Kracke (14). Briefly, after the initial germination stage, cells were permeabilized in RPMI supplemented with 100 μM nigericin and 150 mM KCl and subsequently exposed to extracellular pHs ranging from 4.5 to 7.5. BCECF-AM ratios were then converted to intracellular pH (pH_i_) values using the following equation: pH_i_ = pK_a_ − log[(*R*_max_ − *R*)/(*R* − *R*_min_) × *F*_base436_/*F*_acid436_], where pK_a_ is the acid dissociation constant for BCECF-AM, *R* is the ratio of the emission fluorescence signals measured at 530 nm when the fluorophore is excited at 490 nm and 436 nm, respectively, *R*_max_ is the ratio for maximum fluorescence measured at pH 7.5, *R*_min_ is the ratio for minimum fluorescence measured at pH 4.5, and *F*_base436_/*F*_acid436_ is the ratio of fluorescence signals at 436 nm under the basic and acid conditions used to obtain *R*_max_ and *R*_min_.

## RESULTS

### Acetic acid shows strong antifungal activity against Mucorales species.

**FIG 1:**
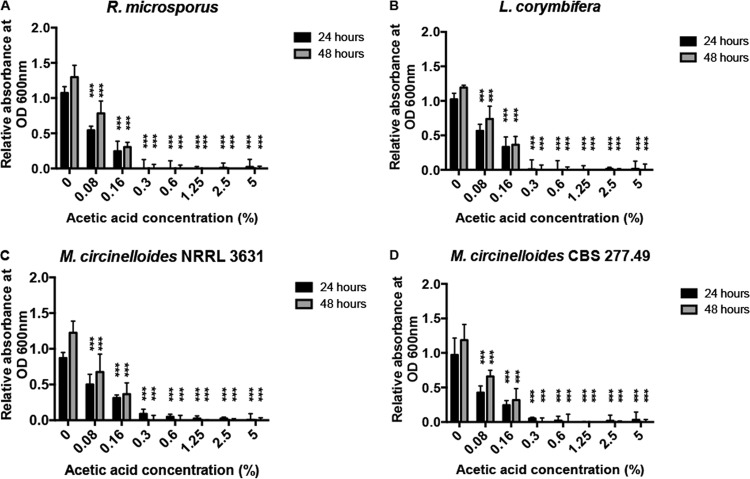
Acetic acid inhibits fungal spore germination. Spores were grown in Sabouraud medium, and growth was assessed by OD_600_ measurements. Graphs show OD measurements after 24 and 48 h. Error bars represent standard deviations (*n* = 4, with three experimental replicates at each time point); statistical analysis was conducted using two-way analysis of variance (ANOVA) with Dunnett posttest. ***, *P* < 0.001.

**FIG 2:**
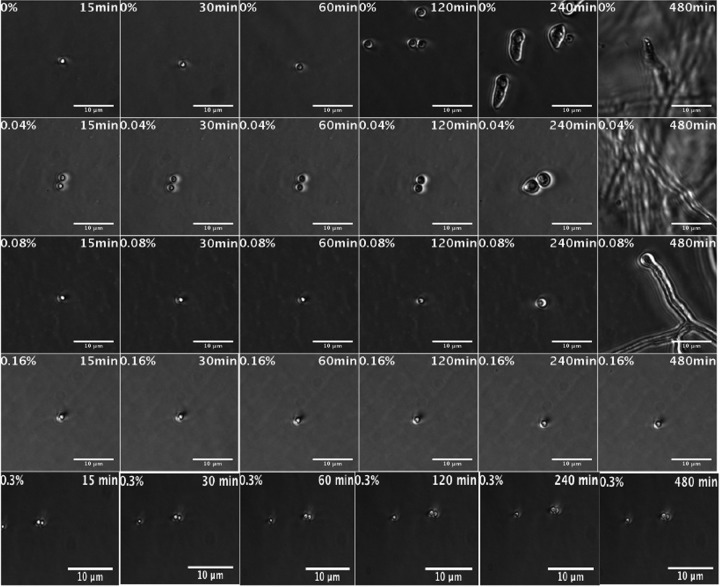
Visualization of acetic acid activity against R. microsporus. Time-lapse imaging shows that acetic acid concentrations of 0.16% or above strongly inhibit germination. Frames are extracted from Movies S1 to S5 in the supplemental material.

Ubiquitous fungal spores are the infectious agent for mucormycosis. Symptomatic mucormycosis depends on germination of fungal spores and subsequent tissue-invasive hyphal growth. To test whether spore germination was inhibited by acetic acid, spores of Rhizopus microsporus 12.6652333, Lichtheimia corymbifera 9.6002134, and Mucor circinelloides NRRL3631 and CBS277.49 (4) were inoculated into Sabouraud broth (to mimic nutrient-rich conditions) and cRPMI (containing serum to test for potentially inhibitory effects) with various acetic acid concentrations. Mucorales grow as filamentous fungi and, thus, OD measurement is problematic for monitoring growth rates but is very successful in monitoring the onset of germination. Acetic acid impaired germination in all isolates at concentrations as low as 0.3% (Fig. 1 and 2; see also Fig. S1 and Movies S1 to S5 in the supplemental material).

**FIG 3:**
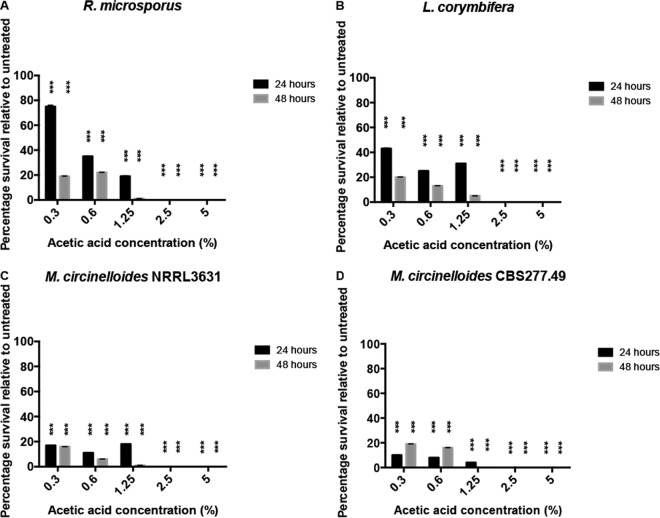
Acetic acid is fungicidal to spores at concentrations of 2.5% and above. Spores were grown in Sabouraud medium and then plated after 24 h of incubation for CFU counts. Graphs show CFU/ml. Error bars represent standard deviations (*n* = 3, with three experimental replicates at each time point); statistical analysis was conducted using two-way ANOVA with Dunnett posttest. ***, *P* < 0.001.

**FIG 4:**
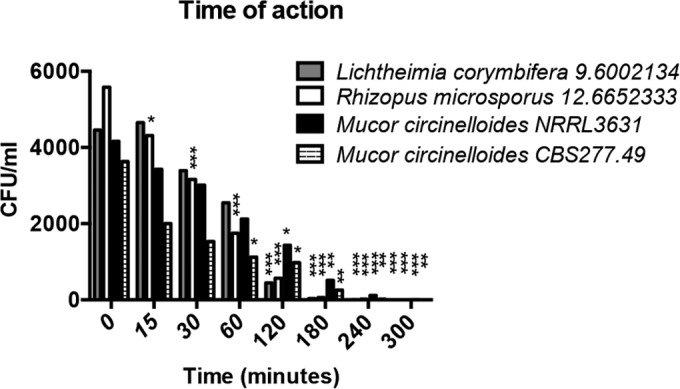
The fungicidal activity of 2.5% acetic acid requires prolonged incubation. Spores were grown in RPMI medium supplemented with 2.5% acetic acid and plated onto Sabouraud agar plates every 15, 30, 60, 120, 180, 240, and 300 min. Numbers of colonies were counted 24 h later. Error bars represent standard deviations (*n* = 3, with three experimental replicates at each time point); statistical analysis was conducted using two-way ANOVA with Dunnett posttest. *, *P* < 0.05; **, *P* < 0.01; ***, *P* < 0.001.

To test whether this effect was fungistatic or fungicidal, spores were plated onto Sabouraud agar plates and the number of colonies was counted after 24 h. At concentrations of 2.5% and above, no viable colonies could be recovered (Fig. 3; see also Fig. S2 in the supplemental material). Time course analysis indicated that this fungicidal activity peaks following approximately 4 h of exposure to 2.5% acetic acid (Fig. 4). Thus, acetic acid strongly suppresses fungal germination at very low concentrations and is potently fungicidal at concentrations above 2.5%.

### Antifungal activity against Mucorales is not seen with other acids.

**FIG 5:**
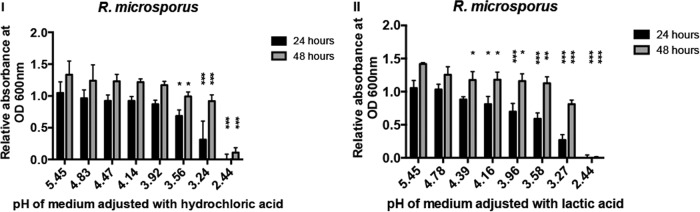
Other acids are less effective at inhibiting spore germination. Neither hydrochloric (I) nor lactic (II) acid was able to inhibit spore germination as effectively as acetic acid. Spores were grown in Sabouraud medium, and growth was assessed by OD_600_ measurements. Graphs show OD measurements after 24 and 48 h. Error bars represent standard deviations (*n* = 3, with three experimental replicates at each time point); statistical analysis was conducted using two-way ANOVA with Dunnett posttest. *, *P* < 0.05; **, *P* < 0.01; ***, *P* < 0.001.

To investigate whether this effect is driven solely by environmental pH, we performed similar experiments using an alternative organic acid (lactic acid) and an inorganic acid (hydrochloric acid). We matched the pHs of Sabouraud medium seen at different concentrations of acetic acid with both hydrochloric and lactic acid (Tables 1 and 2) and monitored the cultures for spore germination. Neither hydrochloric nor lactic acid (Fig. 5; see also Fig. S3 to S5 in the supplemental material) significantly inhibited spore germination other than at the very lowest pHs. Thus, the antifungal effect of acetic acid is not simply a reflection of lowered environmental pH.

### Acetic acid inhibition of spore germination is not solely due to reduced intracellular pH.

**FIG 6:**
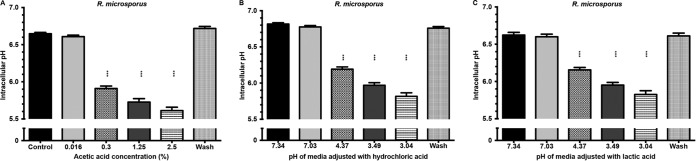
Intracellular pH measurements following treatment with different acids and, following experimentation, upon return to neutral pH via a wash with buffered medium. Spores were grown in RPMI medium supplemented with different acids. The intracellular pH was measured using the calibrated ratiometric analysis of the BCECF-AM dye described in Materials and Methods. Error bars represent standard errors of the means (*n* = 3, with three experimental replicates at each time point); statistical analysis was conducted using two-way ANOVA with Dunnett posttest. ***, *P* < 0.001.

To investigate the mechanism by which acetic acid inhibits spore germination, we measured the intracellular pH of fungal spores during exposure to acetic, hydrochloric, and lactic acid. Cells were incubated with the pH-responsive BCECF-AM dye and monitored following acid exposure. At identical extracellular pHs, acetic acid lowered intracellular pH more strongly than hydrochloric or lactic acid, likely due to stronger dissociation of the ions within the fungal cytoplasm (Fig. 6; see also Fig. S6 in the supplemental material). However, even at identical intracellular pHs, acetic acid is far more effective at inhibiting fungal germination, suggesting that this inhibition is not solely due to the raised intracellular hydrogen ion concentration (e.g., compare growth inhibition by 0.3% acetic acid in Fig. 1 with growth inhibition by hydrochloric acid at pH 3.56 in Fig. 5, both of which drive an intracellular spore pH of 5.9, as shown by the results in Fig. 6).

### Growth inhibition by acetic acid involves both pH- and acetate-dependent effects.

**FIG 7:**
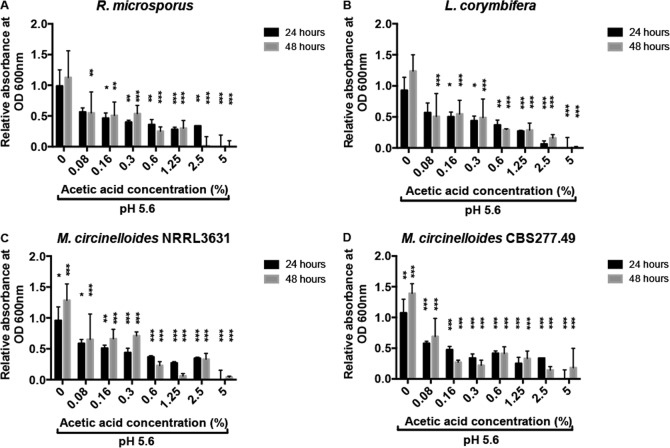
Neutralized acetic acid inhibits fungal spore germination with moderate efficacy. Spores were grown in Sabouraud medium, and growth was assessed by OD_600_ measurements. Graphs show OD measurements after 24 and 48 h. Error bars represent standard deviations (*n* = 4, with three experimental replicates at each time point); statistical analysis was conducted using two-way ANOVA with Dunnett posttest. *, *P* < 0.05; **, *P* < 0.01; ***, *P* < 0.001.

To test a potential pH-independent effect of acetic acid, we neutralized different concentrations of acetic acid by adding NaHCO_3_ to return the pH to that of the control but retain the presence of free acetate. These pH-neutralized media continued to inhibit spore germination (Fig. 7), less effectively than nonneutralized acetic acid but more effectively than other acids. Thus, the suppression of fungal germination by acetic acid involves both pH-dependent and pH-independent but acetate-dependent mechanisms.

### Acetic acid shows activity against actively growing Mucorales.

**FIG 8:**
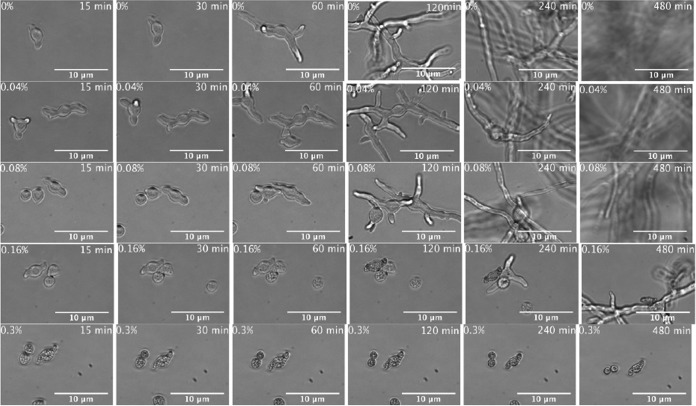
Acetic acid exerts an antifungal activity on pregerminated spores. Time-lapse imaging shows prolonged inhibition of further fungal growth in germinated spores at acetic acid concentrations of 0.3% or above. Time-lapse frames are derived from Movies S6 to S9 in the supplemental material.

In a clinical setting, Mucorales spores may have germinated before treatment can be initiated. Thus, to investigate whether acetic acid is active against germinated spores, we monitored filamentously growing fungal cells by time-lapse microscopy and then exposed them to different concentrations of acetic acid (Fig. 8; see also Movies S6 to S9 in the supplemental material). As with nongerminated spores, growing fungi were strongly inhibited by acetic acid concentrations as low as 0.3% (Fig. 8). Thus, acetic acid is effective even against spores that have already germinated.

## DISCUSSION

Here, we describe the fungistatic and fungicidal properties of acetic acid against several mucormycete species.

Mucormycosis is a life-threating infection, with mortality rates of more than 90%. While susceptible patient groups (e.g., those with impaired immunity and traumatic injuries) are increasing, diagnosis is often delayed and antifungal therapy typically ineffective and expensive. Hence, there is a need for prompt prophylactic treatment for high-risk patients (e.g., severely injured patients). Therefore, we have investigated the efficacy of acetic acid, a traditional method used in medicine, as a topical treatment for this fatal fungal infection.

We here demonstrate that concentrations of acetic acid as low as 0.3% are able to inhibit the spore germination and fungal growth of four isolates, representing diverse Mucorales species (Rhizopus microsporus 12.6652333, Lichtheimia corymbifera 9.6002134, and two isolates of Mucor circinelloides, NRRL3631 and CBS277.49) (4).

Very early studies of acetic acid activity against bacteria by Levine and Fellers (15) suggest that acetic acid toxicity is not due to hydrogen ion concentration alone but seems to be a function of the concentration of undissociated acid. Such a model is supported by our observation that acetic acid is more potently antifungal than other organic and inorganic acids (lactic and hydrochloride acid, respectively), even when matched for extracellular or intracellular pH. Thus, the antifungal activity of acetic acid seems to be a function of both hydrogen ion concentration and undissociated acid, or free acetate. As undissociated acid molecules diffuse through the cell membrane, they dissociate further within the cytoplasm. Thus, the fungus must expend energy both to pump out excess protons and to deal with free acetate within the cytoplasm.

Acetic acid is an abundant and cheap compound that is temperature stable and nonhazardous. It is therefore extremely amenable to use as a topical antifungal in challenging clinical situations, for instance, as a topical wound dressing for military personnel or those working in remote areas where Mucormycete infection is likely. Since it remains highly effective even at low concentrations, there is very limited risk of adverse effects or patient discomfort. Thus, our data suggest that clinical trials to test the efficacy of rapid, topical application of acetic acid are warranted and that this treatment may offer an effective, low-cost prophylactic treatment for an infectious disease that is currently very challenging to treat.

## Supplementary Material

Supplemental material
