# Supplementary material for: pH Manipulation as a Novel Strategy for Treating Mucormycosis
Source: Antimicrob Agents Chemother. 2015 Oct 13;59(11):6968–74. doi: 10.1128/AAC.01366-15 (PMC4604374; doi:10.1128/AAC.01366-15)
Supplement: Supplemental material [file supp_59_11_6968__index.html]

Supplemental material 

# pH manipulation as a novel strategy for treating mucormycosis.

## Supplemental material

- Supplemental file 1 -

  Supplemental Figures S1 to S6

  PDF, 939K
- Supplemental file 2 -

  Supplemental Movie S1: visualization of 0.3% acetic acid activity against *R. microsporus*.

  MOV, 603K
- Supplemental file 3 -

  Supplemental Movie S2: visualization of 0.16% acetic acid activity against *R. microsporus*.

  MOV, 672K
- Supplemental file 4 -

  Supplemental Movie S3: visualization of 0.08% acetic acid activity against *R. microsporus*.

  MOV, 633K
- Supplemental file 5 -

  Supplemental Movie S4: visualization of 0.04% acetic acid activity against *R. microsporus*.

  MOV, 634K
- Supplemental file 6 -

  Supplemental Movie S5: visualization of 0% acetic acid activity against *R. microsporus*.

  MOV, 596K
- Supplemental file 7 -

  Supplemental Movie S6: acetic acid 0.3% antifungal activity on germinating spores of *R. microsporus*.

  MOV, 754K
- Supplemental file 8 -

  Supplemental Movie S7: acetic acid 0.16% antifungal activity on germinating spores of *R. microsporus*.

  MOV, 914K
- Supplemental file 9 -

  Supplemental Movie S8: acetic acid 0.08% antifungal activity on germinating spores of *R. microsporus*.

  MOV, 863K
- Supplemental file 10 -

  Supplemental Movie S9: acetic acid 0% antifungal activity on germinating spores of *R. microsporus*.

  MOV, 708K
