# Supplementary material for: pH Manipulation as a Novel Strategy for Treating Mucormycosis
Source: Antimicrob Agents Chemother. 2015 Oct 13;59(11):6968–74. doi: 10.1128/AAC.01366-15 (PMC4604374; doi:10.1128/AAC.01366-15)
Supplement: Supplemental material [file AAC.01366-15_zac011154533so1.pdf]

Supplementary Figure 1. Acetic acid shows strong antifungal activity in complete RPMI media.

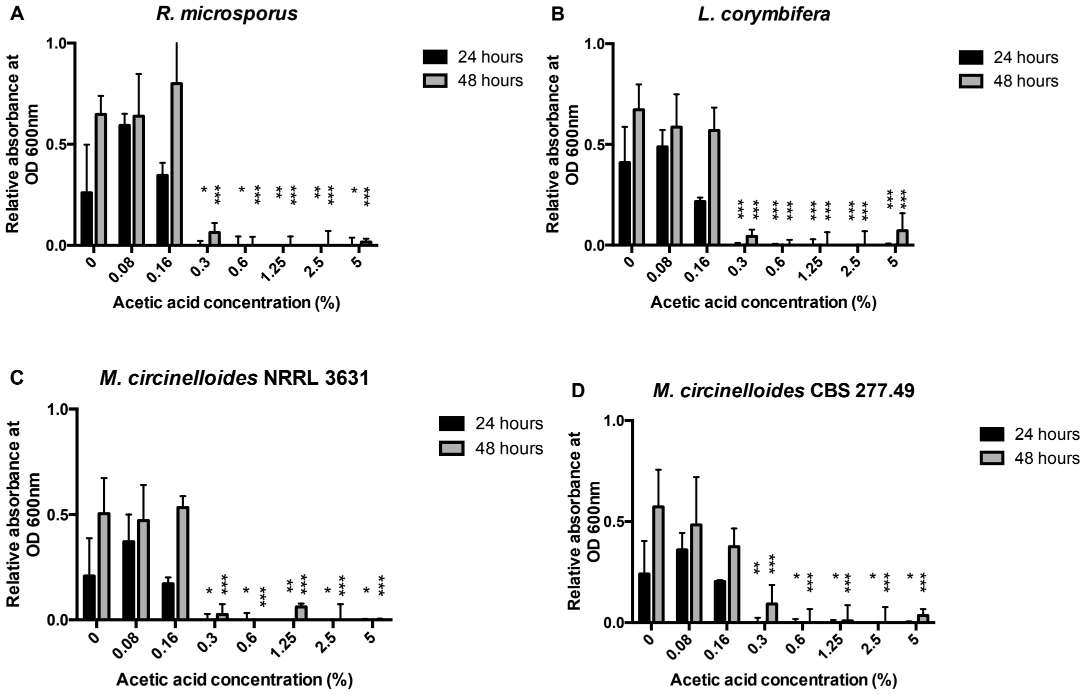

Supplementary Fig. 1. Acetic acid shows strong antifungal activity in complete RPMI media.

Spores were grown in cRPMI media supplemented with different concentrations of acetic acid and spore germination was assessed by OD 600 nm measurements. Graphs show OD measurement after 24 and 48 hours. Error bars represent standard deviation (n=4, three experimental replicates at each time) and statistical analysis was conducted using a two-way ANOVA with Dunnett post-test. . *$p<0.05$, **$p<0.01$, *** $p<0.001$.

Supplementary Figure 2. Fungicidal features of acetic acid in complete RPMI media.

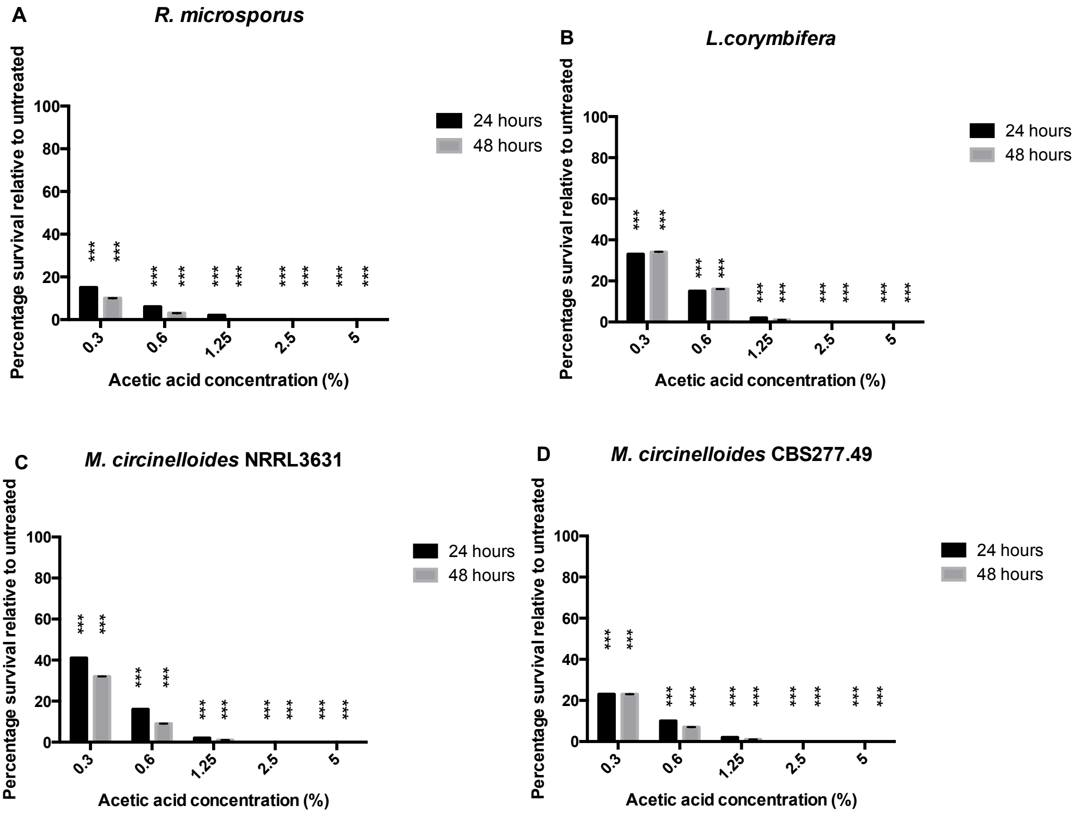

Supplementary Fig. 2. Fungicidal features of acetic acid in complete RPMI media. Acetic acid is fungicidal to spores at the concentrations of 2.5% and above. Spores were grown in cRPMI media and plated after 24 hours for colony forming unit (CFU) counts. Graphs show colony-forming unit per milliliter grown after 24 hours. Error bars represent standard deviation (n=3, three experimental replicates at each time) and statistical analysis was conducted using a two-way ANOVA with Dunnett post-test. Spores were grown in cRPMI media. * $p<0.05$, ** $p<0.01$, *** $p<0.001$.

Supplementary Figure 3. Lack of inhibition of spore germination by other acids.

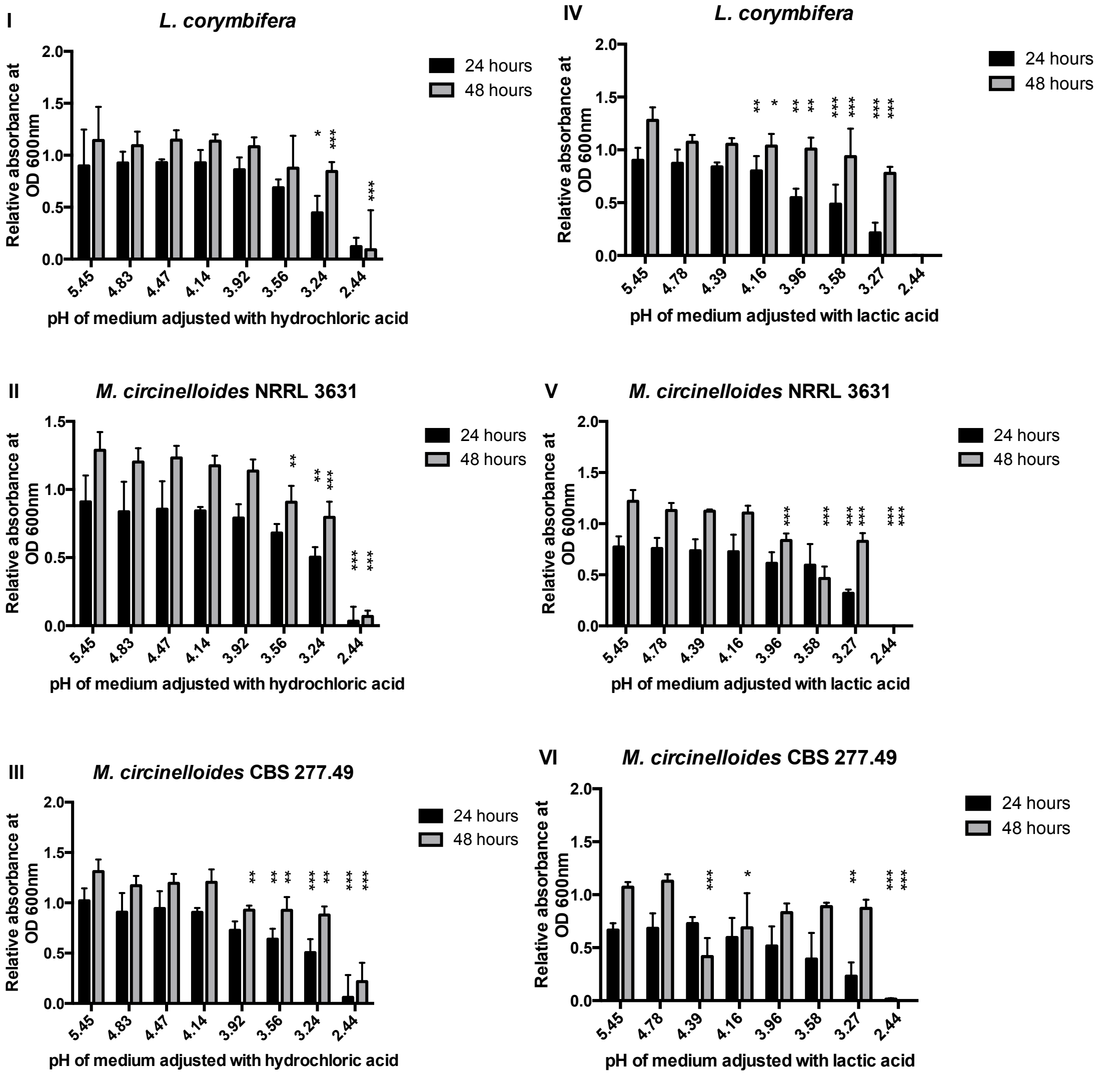

Supplementary Fig. 3. Lactic and hydrochloric acid fail to inhibit spore germination.

Neither hydrochloric (I, II, III) nor lactic (IV, V, VI) acids are able to inhibit spore germination as effectively as acetic acid. Spores were grown in Sabouraud media supplemented with different concentrations of lactic or hydrochloric acid and spore germination was assessed by OD 600 nm measurement. Graphs show OD measurement after 24 and 48 hours. Error bars represent standard deviation (n=3, three experimental replicates at each time) and statistical analysis was

conducted using a two-way ANOVA with Dunnett post-test. *$p<0.05$, **$p<0.01$, *** $p<0.001$.

Supplementary Figure 4. Hydrochloric acid has no effect on inhibition of spore germination in complete RPMI media.

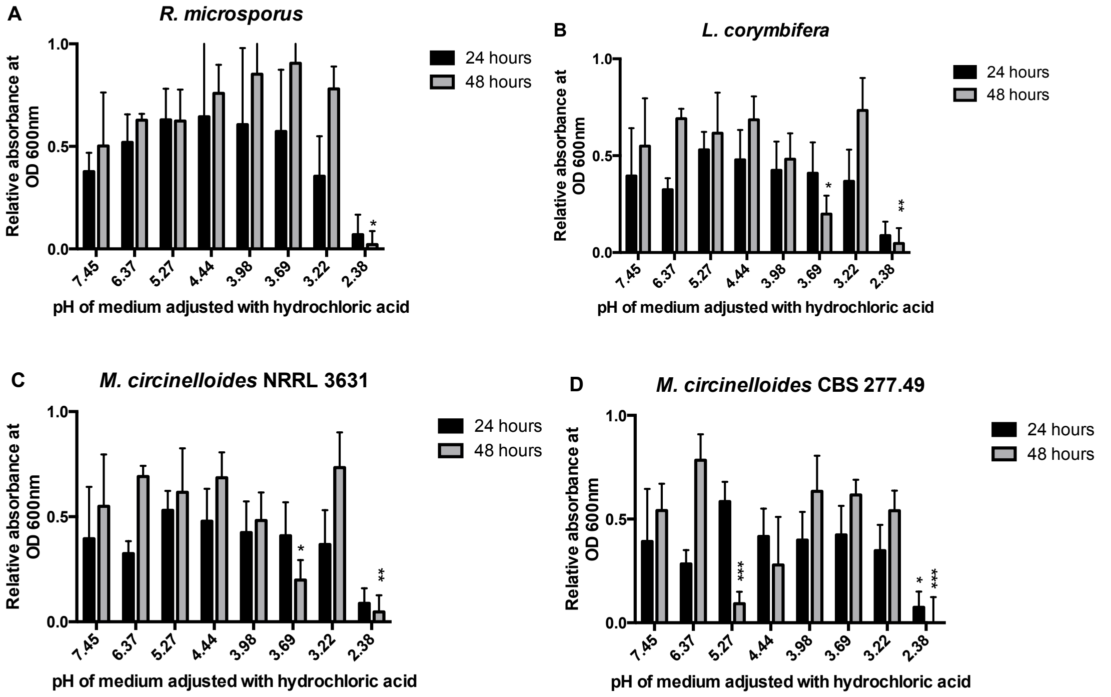

Supplementary Fig. 4. Hydrochloric acid has no effect on inhibition of spore germination in complete RPMI media.

Hydrochloric acid is not able to inhibit spore germination as effectively as acetic acid. Spores were grown in cRPMI media supplemented with different concentrations of hydrochloric acid and spore germination was assessed by OD 600 nm measurements. Graphs show OD measurement after 24 and 48 hours. Error bars represent standard deviation (n=3, three experimental replicates at each time) and statistical analysis was conducted using a two-way ANOVA with Dunnett post-test. * $p<0.05$, **$p<0.01$, *** $p<0.001$.

Supplementary Figure 5. Lactic acid does not inhibit spore germination in complete RPMI media.

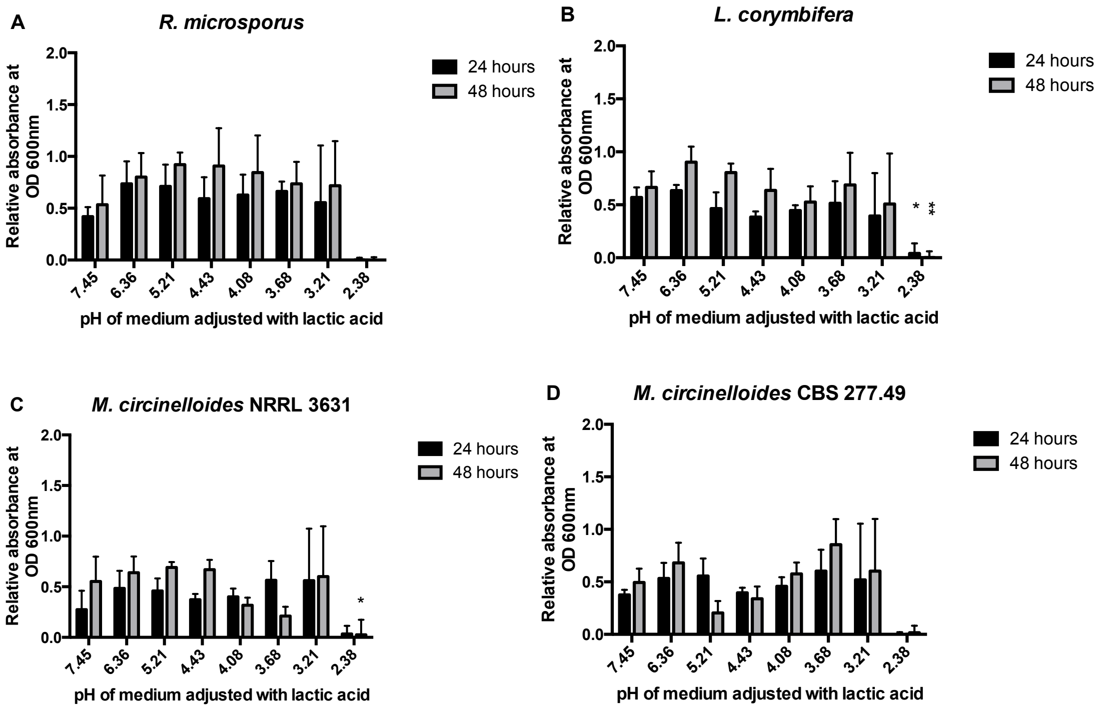

Supplementary Fig. 5. Lactic acid does not inhibit spore germination in complete RPMI media.

Lactic acid is not able to inhibit spore germination as effectively as acetic acid. Spores were grown in cRPMI media supplemented with different concentrations of lactic acid and spore germination was assessed by OD 600 nm measurements. Graphs represent OD measurement after 24 and 48 hours. Error bars represent standard deviation (n=3, three experimental replicates at each time) and statistical analysis was conducted using a two-way ANOVA with Dunnett post-test. * $p<0.05$, ** $p<0.01$, *** $p<0.001$.

Supplementary Figure 6. Intracellular pH is lowered more effectively by acetic acid at the same extracellular pH.

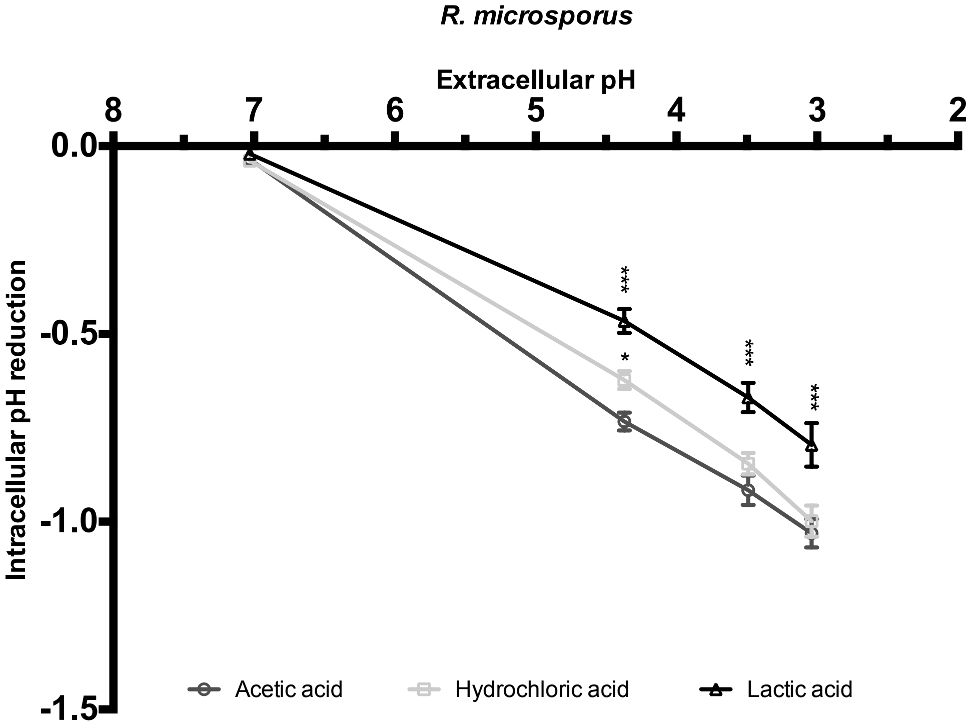

Supplementary Figure 6. Intracellular pH is lowered more effectively by acetic acid at the same extracellular pH.

Data shown are derived from the same experiment as Figure 7. Spores were grown in RPMI media supplemented with different acids. The intracellular pH was measured using the calibrated ratiometric analysis of the BCECF-AM dye, as described. Error bars represent standard error of the mean (n=3, three experimental replicates at each time) and statistical analysis was conducted using two-way ANOVA with Dunnett post-test. *$p<0.05$, **$p<0.01$, ***$p<0.001$
